# The Varioloid Disease

**Published:** 1838-09-12

**Authors:** N. Chapman

**Affiliations:** Professor of the Theory and Practice of Physic, in the University of Pennsylvania


### THE MEDICAL EXAMINER.

DEVOTED TO MEDICINE, SURGERY, AND THE COLLATERAL SCIENCES.

EDITED BY J. B. BIDDLE, M. D. AND M. CLYMER, M. D.

No. 19.] PHILADELPHIA, WEDNESDAY, SEPTEMBER 12, 1838. [Vol. I.

### THE VARIOLOID DISEASE.

*By* N. Chapman, M. D., *Professor of the Theory and Practice of Physic, in the University of Pennsylvania.*

[Reported for this Journal.]

What were the struggles and difficulties with which vaccination had to contend in the commencement, and how slow and reluctant were the concessions to its validity and usefulness, are sufficiently known. To receive, at once, a discovery so novel in itself, of such high pretensions, and opposed, as it was, by the whole tenor of analogy and experience, could not reasonably be expected. It was, accordingly, subjected to the severest ordeal its enemies could suggest; and, in this scrutiny, objections were removed, prejudice after prejudice worn away, till conviction took the place of doubt and hesitation, and its triumph was signal and complete.

During many years, this state of unqualified confidence continued unabated, in every enlightened section of the world. Now and then, it is true, an instance of failure would occur, from causes not at all to impeach its general power of protection, by which some clamour against it was raised, and some temporary popular distrust created. The profession, however, with one or two immaterial exceptions, seemed insensible to such impressions, and remained, with wonderful consistency, steadfast in their faith. Confiding, indeed, too implicitly, our discernment to real imperfections became impaired, and even a disposition approaching to intolerance arose, which made it reproachful to seem incredulous, much less to express doubts as to the infallibility of the process. But, now, under a sort of panic terror, we are hastening into an opposite extreme, prepared to confess our errors, and renounce that practice which had been cherished and defended, with the vehemence and intensity of blind devotedness. Can such a course be reconciled to what is due to our dignity, to the cause we have heretofore so warmly espoused, or to that community which looks up to us to guide them in this momentous concern, by our science and deliberative wisdom? Every obligation, properly operative in the case, directs us to pause, to retrace calmly our steps, and dispassionately to survey the whole subject, to contemplate it in its various lights, to contrast its merits with its demerits, and studiously, by every help, to endeavour to arrive at a just decision.

That vaccination has sometimes proved inefficient, is not denied even by its warmest advocates. To this point evidence has, by degrees, accumulated, of such force and certainty, as not to be resisted.

The sources of many of these failures, I have in a preceding lecture fully developed, and shown that, for the most part, they are under our control, and, as proceeding from ignorance or negligence, may, by proper care, be hereafter avoided.

In relation to the subject, such was pretty generally the sentiment, when a disease broke out in Europe, which has led to some new views, and, ultimately, as must be confessed, to a much lower estimate of vaccination than formerly.

This disease seems to have prevailed for the first time to any extent at Edinburgh, in the winter of 1818. It had previously shown itself in several provincial towns of Scotland; and though exciting some curiosity, commanded no serious attention. Contemporaneously, or nearly so, it raged in England, particularly at Norwich, as well as on the Continent, in France, at Geneva, in Italy, in Holland, and Germany.

Emanating from these points, it progressively spread throughout Europe, scarcely any one portion of it escaping. Crossing the Atlantic, it appeared the next year among us, diffusing itself over the United States, thence into Mexico, South America, and the Antilles—subsequently broke out in the East Indies, and seems in a greater or less degree to have pervaded the world, affording an instance of one of the most extensive epidemics on record.

It is described by the foreign writers as assailing three classes of persons,—those who had passed through small-pox naturally or artificially—those who had been vaccinated, and those who had been subjected to neither of these processes.

By Professor Thompson, of Edinburgh, an elaborate treatise has been published, embracing a formal and systematic history of the disease. Declining, for obvious reasons, to follow him in detail, I shall attempt to present, in a mere summary, some of the leading and most important of his matter.

In persons having had neither small-pox nor cow-pox, the eruption is represented as preceded by fever, commonly of great violence, though sometimes comparatively moderate, continuing for three days, and eventuating in a variolous eruption of various gradations of severity. Of two hundred and five persons, whom he saw in this form of the epidemic, fifty died, making a proportion of one in four, which, independently of other evidence, shows its violent and uncontrollable nature. In those who had previously had small-pox, the eruptive fever, in very many, was severe, and in others, so mild, as scarcely to be perceptible. The eruption, for the most part resembled the chicken-pock in its several varieties, though in some instances it had the appearance either of the discreet or confluent small-pox. Of this form of the disease, he saw or heard of seventy-one cases, three of which died, giving the proportion of one in twenty-three. It is worthy of remark, that in two of the fatal cases, the attack recurred in a few weeks after having had small-pox.

The disease in persons previously vaccinated, seems not to have differed materially, from that under the immediate preceding circumstances. Describing it, he indeed employs nearly the same language, though on the whole, it may, I think, be collected, that such cases were milder. Even when the fever was ever so violent, it almost uniformly ceased on the appearance of the eruption. Now and then, however, it assumed the shape of some of the worst species of small-pox, and ran a protracted course. It would be very interesting to determine, whether the system in these inveterate cases, had been protected by vaccination faithfully done. We are now aware to how many contingencies is that process exposed. Be this as it may, it is consolatory to learn, that of three hundred and ten individuals affected after vaccination, only one perished, and whose death can hardly be ascribed to this cause. Of the above number, forty had the disease a second time,—only a single instance is mentioned of its returning a third time, and repetitions of attack were distinguished by no peculiarities. It is observable that a large proportion of those who were seized with the disease, after vaccination, had been in the intervals inoculated with small-pox, or exposed to its agency, without being affected. From the accounts in my possession, there was such an essential uniformity as it prevailed generally abroad, that what I have stated may suffice to convey an adequate notion of the epidemic.

As it appeared in various sections of the United States, my knowledge is not very precise. The accounts, however, which were furnished me at the time, by two of my correspondents at Lancaster in this state, where it first occurred, show it in an aspect very different from its exhibition in Europe. It broke out in November, 1818, and was alleged to be traced to some German emigrants who disseminated it in passing through that city into the interior of the country. The disease, it is true, attacked indiscriminately the variolated, the vaccinated, and unprotected, though not in the same proportions. Of the first description, or those who had previously had small-pox, there were six cases, of whom none died—of the second, or vaccinated, forty, two of whom, very young children, died in convulsions—and of the third, or unprotected, three hundred and fifty, among whom there were four deaths. This slender mortality, with some other facts, lead me to suspect that the disease was varicella. It may be remarked particularly, in confirmation of this suspicion, that chicken-pock seems every where to have preceded or accompanied the more formidable epidemic.

From Baltimore, where it prevailed in the winter of 1821, and still more violently in 1822, my intelligence is still more defective. It is stated, however, to be the common impression, that it was imported from Liverpool—though this is doubted—and we learn that it occasionally attacked both the vaccinated and variolated, I presume in a mitigated shape, since no death occurred under such circumstances. The unprotected suffered much, many being affected, attended with a mortality of about one in six or seven cases. This is the substance of various communications which have reached me, separated from a mass of vague and contradictory statements.

In relation to the disease in this city, so early as June, 1823, an eruptive fever, which was considered as ordinary varicella, made its appearance, and spread pretty extensively. Contemporaneously prevailed also scarlatina, rubeola, and erysipelas, with a variety of anomalous cutaneous affections. Measles, especially, was very rife, and generally of a highly exasperated character, so that former attacks of the disease, in several instances, afforded no protection. During the existence of these exanthemata, some time in July, four cases of very strongly variolous character occurred, nearly at the same time, in widely separated parts of the city, the origin of which could not be traced to any source of contagion. Cases of this description generally multiplied, and by the commencement of November they had become numerous, though almost exclusively confined to Southwark, among the poorest class of our population.

It was about this period, that some alarm was excited by the occasional occurrence of it in persons who had been previously vaccinated, and such failures daily increasing, no doubt was longer entertained, that the same epidemic which elsewhere produced so much solicitude, had broken out among us. The disease henceforward ran its course, and in most of its features, conformed to what has been observed in regard to it in other places. It attacked the variolated, the vaccinated, and unprotected, occasioning those modifications under the several circumstances stated, which are so accurately described by the foreign writers. As far, indeed, as came under my own immediate observation, there was no material difference. Every degree was presented from that of the mildest varicella to the most malignant small-pox. Generally it was of the former character. Commencing with a slight fever, which endured from one to three days, the eruption appeared, sometimes merely an efflorescence, though usually as minute papulæ, many of which speedily dried away, while others ran on to the formation of vesicles or pustules. The latter were of diverse shapes, conoidal, lenticular, oval, circular, flattened on the surface, with a central depression, and an imperfect areola around some of them, bearing, on the whole, a resemblance more or less to the vaccine or variolous pustules. Great difference existed as to the extent of the eruption, and the manner in which it came forth, in some instances confined, and that very sparsely to the head,—in others, the whole body was covered pretty thickly, breaking out simultaneously, or in successive crops, occasionally of one uniform character, or exceedingly diversified, being papular, vesicular, or pustular, &c. Commonly, all febrile excitement subsided on the manifestation of the eruption; and copious as this might be, it was not followed by any secondary fever. The eruption rapidly faded away. Even when consisting of pustules, these began to desiccate in two or three days, into thin darkish scabs, which soon after fell off, leaving a smooth florid surface, with, perhaps, here and there, a pit or indentation, or a small fungoid excrescence. But this account regards only the benignant form of the affection, in its several gradations. As intimated, it sometimes exhibited a much more formidable aspect, having every feature, in its progressive stages, of discreet or confluent small-pox, and under circumstances, too, of an antecedent subjection of the system to variolation or vaccination by the most skilful practitioners. Taking place without the mitigating influence of these processes, it was nearly always, apparently, variola, and generally, in its most malignant typhoid shapes, proving as incurable as probably ever was known. But in some particulars the epidemic differed, and among which the protective powers of vaccination proved with us infinitely less than elsewhere. From data tolerably authenticated, it is computed that between four and five thousand failures of this process took place; and I have not been able to collect more than thirty instances of secondary small-pox, and very few, where the previous attack was in the natural way, or so violent by inoculation as to have left any marks behind.*

The following table, taken from the report of Drs. Mitchell and Bell, who had charge of the small-pox hospital, is interesting in several views. It furnishes a statement of the result of one hundred and forty-eight cases of the disease:

"There were forty-seven cases in persons who had been previously affected by vaccination, none of whom died. Eight cases occurred in persons previously affected with small-pox, of whom four died and four recovered. Ninety-three cases were in persons who had not had either disease before, of whom fifty-two died, and forty-one recovered.

"Of the whole number, sixty-nine were whites, and seventy-nine persons of colour. Two, out of the eight persons, who had small-pox a second time, took it the first time naturally, or without inoculation. Eight of those vaccinated were so during the prevalence of the epidemic, and some of the mildest cases were in the persons of those who had been vaccinated upwards of twenty years before."

This table includes the results only to the 14th of January. The relative proportions, however, subsequently, in every respect, were pretty nearly the same.

Thé record of the *Board of Health* up to the same time, shows that little more than three hundred died of small-pox, and four only of the varioloid disease; whether the last followed variolation or vaccination, does not appear.

No instance, at this period, or for several years afterwards, came within my knowledge of any repetition of attack in the same person, as noticed in Europe. But subsequently, I have seen it in three families to the amount of seven cases.

It may here be remarked, that the disease, in conformity with most others dependent on a specific contagion, gradually declined on the accession of warm weather, and by the 1st of June entirely subsided. The next winter, however, it reverted, though sparsely, and thence ceased, with perhaps here and there a separate case, till the succeeding winter, when it again returned pretty much in the same manner as before. From 1825 to 1827, so little was heard of it that hopes were entertained of its total disappearance, when it once more revisited our city to a considerable extent. During the next two years, it became nearly extinct, and so continued till the winter of 1830, on which occasion, numerous cases recurred. Not much was heard of it after that period, though occasionally solitary instances were met with. But in 1833, it again revived and spread widely, since which, we have had scarcely any of it, and probably the epidemic has become exhausted. Each of its renewals have been marked by pretty nearly the same phenomena, varied chiefly by gradations of violence, and in every instance preceded by varicella, scarlatina, rubeola, as well as by an infinity of anomalous eruptions, and other cutaneous affections. Do not suppose that we have been exclusively the victims of this disease. Nearly all our cities, and many portions of the country, have been exposed to its ravages, during the same period, though excepting, perhaps, New York, not in the same degree.

(To be continued.)

* It is a very curious fact, that neither Dr. Physick nor myself, on this or on any other occasion, ever met with an unequivocal instance of secondary small-pox. Many of the cases reported to be such I visited, and detected a source of deception which ought to be guarded against in the investigation of the subject. The disease chiefly prevails among the most stupid and ignorant classes of society, by whom the term inoculation is only employed, and hence they are apt to report themselves as having been variolated, when really the act was that of vaccination.

